# Obstetric Violence in Spain (Part III): Healthcare Professionals, Times, and Areas

**DOI:** 10.3390/ijerph18073359

**Published:** 2021-03-24

**Authors:** Desirée Mena-Tudela, Susana Iglesias-Casás, Víctor Manuel González-Chordá, María Jesús Valero-Chillerón, Laura Andreu-Pejó, Águeda Cervera-Gasch

**Affiliations:** 1 Department of Nursing, Faculty of Health Sciences, Universitat Jaume I, Avda. Sos I Baynat s/n, 12071 Castellón, Spain; vchorda@uji.es (V.M.G.-C.); chillero@uji.es (M.J.V.-C.); pejo@uji.es (L.A.-P.); cerveraa@uji.es (Á.C.-G.); 2 Department of Obstetrics, Hospital do Salnés, Villgarcía de Aurousa, 36619 Pontevendra, Spain; matronasu@gmail.com

**Keywords:** obstetric violence, Spain, midwife, sexual and reproductive health

## Abstract

Background: Obstetric violence is a worldwide public health problem, which seems greater in Spain. As no studies were found that identify the most representative healthcare professionals, times, and areas involved in obstetric violence, the objective of this work was to study at what time of maternity, with which professionals, and in what areas women identified obstetric violence. Methods: This descriptive, retrospective, and cross-sectional study was performed from January 2018 to June 2019. The main variables were the area (hospital, primary care, both), the time (pregnancy, birth, puerperium), and the professionals attending to women. Results: Our sample comprised 17,541 participants. The area identified with the most obstetric violence for the different studied variables was hospitals. Women identified more obstetric violence at time of birth. Findings such as lack of information and informed consent (74.2%), and criticism of infantile behavior and treatment (87.6%), stood out. The main identified healthcare professionals were midwives and gynecologists, and “other” professionals repeatedly appeared. Conclusions: Having identified the professionals, times, and areas of most obstetric violence in Spain, it seems necessary to reflect on not only the Spanish National Health System’s structure and management but also on healthcare professionals’ training.

## 1. Introduction

Several definitions of the obstetric violence (OV) concept exist, but no international consensus about it has been reached. One definition refers to “any medical practice or attitude expressed by language or actions which, during the gyneco-obstetric follow-up of pregnant women, women giving birth or breastfeeding women, ignores women’s and infants’ rights, desires, decisions, needs, emotions and/or dignity” [1]. This concept contains elements that are closely related to the definition of the positive birth experience that the World Health Organization (WHO) offers. It indicates that a positive birth experience includes giving birth to a healthy baby in an environment free of clinical and psychological risks, and receiving emotional support. It also includes friendly and technically competent clinical personnel, and it is still a positive birth experience even when medical interventions are desired or necessary [2].

So it would seem that OV, the birth experience, and satisfaction with the received healthcare are closely linked. It must be stressed that the negative feelings deriving from these concepts while giving birth may have short- and long-term effects on women’s physical, sexual, and psychological health, and they also have consequences for the relationship with newborn infants [3,4]. Some negative healthcare consequences include postpartum depression, post-traumatic stress disorder, not adapting well to the maternal role and breastfeeding problems, or they may affect women’s desire to have more children [5,6,7,8]. The healthcare professionals who perform or witness OV also produce health consequences, of which secondary traumatic stress and compassion fatigue [9] are the most outstanding disorders, and they can even lead professionals to abandon their profession [10]. Former studies have reported on OV in some countries. In Ethiopia, 75.1% [11] of women report being a victim of OV, with 18.3% in Brazil [12], 28.8% in India [13], 21.2% in Italy [14], or 38.3% in Spain These distinct results could lie in the differences found in each study’s sample sizes, and in discrepancies with the various instruments used to assess OV [15]. Nonetheless, it seems plausible to reflect on the fact that a major healthcare problem exists in the maternity area in healthcare services worldwide. 

When we refer to OV, we apply a broad concept that includes verbal, physical, psychological and sexual violence, social discrimination, negligence in healthcare, and healthcare professionals’ improper use of procedures and technologies [16,17]. Some countries, especially Latin American, have passed specific legislation against OV [18]. However, no such legislation exists in Europe, and OV is a theme that is increasingly becoming the center of debate, particularly by social organizations and movements to defend human rights [19]. In this context, it would seem that most of the medical community does not accept the notion that OV exists. This is proven by professionals’ reactions to how OV is dealt with because they classify it as a criminal concept that is morally inadequate and scientifically unacceptable in Spain [20]. Moreover, healthcare professionals reacted after a scientific publication in Italy that described the OV phenomenon as unrealistic [21]. Actually, the structural characteristics of such violence [9] means that professionals frequently exercise it without realizing it, and it has even become a standard practice [22,23]. This means that without actually perceiving it, some healthcare professionals have acquired an authoritarian role marked by pseudoscientific guidelines (not based on up-to-date evidence), and one based on unequal treatment to stick to comply with the protocols established by health centers. [15,16]. 

Moreover, the birth perception might be somewhat subjective and is influenced by several factors such as women’s sensitivity (in relation to mood, humor, disposition, frame of mind, and company kept), or their way of facing the birth experience (such as personal beliefs, reactions, emotions, reflection) [8,24]. It is necessary to stress that trusting midwives and the whole healthcare team also shape most of this perception [8,24], with a higher level of satisfaction and better neonatal outcomes if midwives are the professionals who attend births [25]. In fact, a recent review of birth satisfaction scales reports that the most studied dimension of all questionnaires is satisfaction with healthcare professionals in relation to care and support, including professional healthcare elements such as human resources availability, perceived healthcare professionals’ competence, emotional support, perceived professional care, and satisfaction with certain professionals such as midwives or gynecologists, professionalism, or empathy in healthcare [26]. Other studies have also indicated that the degree of satisfaction is connected to the personnel’s characteristics, such as taking a friendly and professional attitude, respecting women’s requirements, and healthcare teams being capable of helping women participate in healthcare choices [8].

We found no robust scientific literature that identifies the most prevalent women’s sexual and reproductive healthcare professionals related to OV by women internationally. The two studies we found refer only to a single healthcare center with a relatively small female sample, and both focus on Venezuela, where women identified obstetricians, anesthetists, and female nurses as the main perpetrators of OV while women gave birth [27,28]. Nor did we find any studies that identify the area (primary care or hospital care) where, or the time (pregnancy, birth or puerperium) when, more OV takes place. Most studies refer to the time of giving birth and the hospital area [16].

As pregnancy, birth, and maternity are frequent reasons to access healthcare services, it is necessary to evaluate the care that women receive during these periods. Hence, the objective of the present study was to study which professionals, which areas, and at what times women identify OV during maternity.

## 2. Materials and Methods

### 2.1. Design, Population and Sample

A descriptive, retrospective and cross-sectional study was conducted between January 2018 and June 2019. The followed methodology is set out in detail in the previous publications Part I and Part II [15,29]. Women attended to for pregnancy, childbirth, or abortion during the 2009–2018 period, and who completed the questionnaire, were included in the study. In these previous studies, it was noted that some practitioners’ behaviors were not perceived as OV by women [15,29]. However, these behaviors really occurred, and analyzing who performed them and at what time is interesting.

The present work analyzes a subsample of women who gave birth in Spain during the study period, and it affirmatively answered the questions about not receiving information or giving informed consent, receiving criticisms about their behavior and being treated with childish diminutives, having difficulty in resolving fears, doubts, or concerns, undertaking unnecessary interventions, and lack of postpartum support. The exclusion criteria were giving birth at home or at a hospital outside Spanish territory, and not completing 80% of the questionnaire or more. Women from the Spanish Autonomous Communities (SAC) of Ceuta and Melilla were excluded because they were poorly representative. Those women who did not respond to the SAC items were also excluded.

The study was designed in accordance with the principles of the Declaration of Helsinki (charity, no maleficence, autonomy, and justice) and with Spanish Organic Law 03/2018 on Protection of Personal Data and Guaranteeing Digital Rights. No personal data, IP address, or email that could compromise participants’ identity were collected, and answering the questionnaire implied giving consent. Participants were informed about these aspects before voluntarily answering the questionnaire.

### 2.2. Data Collection

Data collection was carried out between February and April 2018 using an online questionnaire. The questionnaire was sent to healthcare professionals, child rearing groups, breastfeeding support groups, administrators of blogs, and the association Birth is Ours [30], by sending the questionnaire link via social networks such as WhatsApp or Facebook [31,32]. 

The main study variables were information received about the process they experienced and informed consent; being criticized about their behavior; being treated with childish diminutives; not being able to explain fears/doubts; perceived unnecessary interventions; respecting birth plans; supporting baby care; and supporting breastfeeding. These variables were measured as: Yes, No, Do not know/No answer. 

Other variables were added that were related to the professionals practicing OV (gynecologist, midwife, female nurse, auxiliary nurse, pediatrician, anesthetist, other), the times when OV was practiced (while pregnancy, while giving birth, during the puerperium), and the areas it took place in (primary care and hospital). The participants could answer more than one item. The cluster groups that classified SAC were also added according to women’s perceived OV as set out in Part I of this research [15]: type of care received (public, private, or mixed healthcare) and perception of suffering OV. Distribution into cluster groups was as follows: Group 1 was formed by SAC Madrid, Basque Country, Principality of Asturias, and Castilla y León; Group 2 was made up of SAC Catalonia, Valencian Community, Aragón, and Castilla-La Mancha; Group 3 comprised SAC Andalusia, Balearic Islands, Canary Islands, and Navarre; Group 4 consisted of SAC Murcia Region, Galicia, Extremadura, and Cantabria; the last group included only one SAC: La Rioja. 

### 2.3. Statistical Analysis

Data were processed with the Statistical Package program for Social Sciences (SPSS) v. 25, IBM, Armonk, NK, USA. A descriptive analysis of all the variables was performed with frequency and percentage. A bivariate analysis was carried out by a Chi-squared test using contingency tables to compare the main professionals, times, and areas related to OV with the studied variables (information received about the experienced process; criticized about their behavior; treated with childish diminutives; not being able to explain fears/doubts; undergoing unnecessary interventions; respecting birth plans; breastfeeding support). The relation of all these variables with the cluster groups, type of care received, and having perceived OV was also studied. The statistical level of significance was set at *p* < 0.05.

## 3. Results

In all, 17,742 questionnaires were obtained, of which 201 were eliminated (1.13%): 88 (0.49%) because they were completed by women who had given birth abroad or because they were not properly filled in; 17 (0.09%) because they belonged to the SAC Ceuta and Melilla; 96 (0.54%) because they did not include an answer about the province variable. The final sample included 17,541 questionnaires.

Our cluster groups were as follows: 39.0% (*n* = 6849) of our sample corresponded to Group 1; 29.2% (*n* = 5120) corresponded to Group 2; 16.2% (*n* = 2848) corresponded to Group 3; 15.0% (*n* = 2630) corresponded to Group 4; and 0.5% (*n* = 94) corresponded to Group 5. As for type of care received, 65.3% (*n* = 11,450) of the women chose public healthcare, 10.4% (*n* = 1830) chose private healthcare, and 24.3% (*n* = 4261) chose mixed healthcare. Finally, 38.3% (*n* = 6051) of our participants affirmatively answered the question about suffering OV [15]. 

### 3.1. Informed Consent Requested and Information Received in Relation to Healthcare Professionals, Times, and Areas

Of the complete sample, 45.9% (*n* = 8047) answered that they were neither informed about the procedures they were about to undergo nor expressly requested to provide informed consent. Of these, 51.8% (*n* = 4150) identified midwifes as the professional responsible for not informing them or requesting their informed consent, 74% (*n* = 5927) indicated gynecologists, 29.9% (*n* = 2395) indicated nurses, 13.1% (*n* = 1052) indicated pediatricians, and 15.7% (*n* = 1259) indicated anesthetists. For 3.9% (*n* = 312), professionals were “other”. Lack of consent or information took place during pregnancy for 38.2% (*n* = 3051) of cases, while giving birth for 81.0% (*n* = 6473) and during the puerperium for 20.7% (*n* = 1653). The area most indicated for lack of informed consent and information was hospitals for 81.5% (*n* = 6526) of all cases.

**Figure 1:**
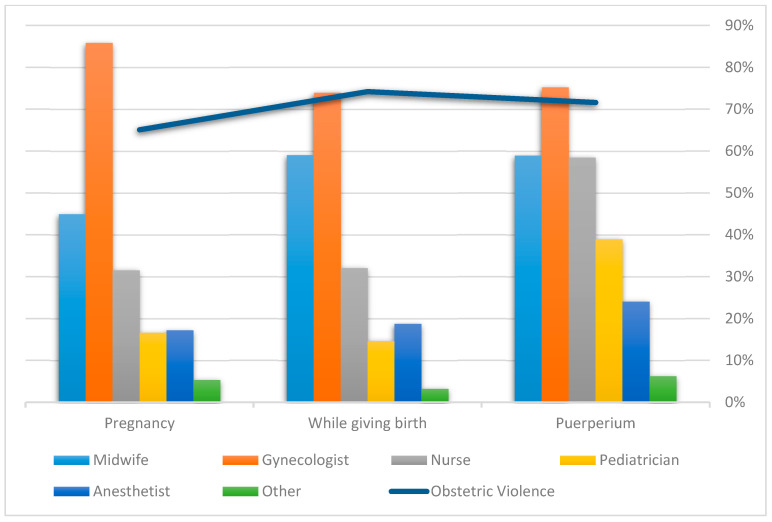
The most representative professionals at all the times care was received in relation to informed consent requested and information received.

On received information and informed consent requested during procedures, the bivariate analysis showed statistically significant differences for type of care received (Public: Yes = 57.2%, *n* = 5197; No = 74.7%, *n* = 6014; Private: Yes = 8.0%, *n* = 724; No = 13.3%, *n* = 1068; Mixed: Yes = 34.9%, *n* = 3172; No = 12.0%, *n* = 968; *X*^2^ = 1248.310, df = 4; *p*-value < 0.001) and perceiving OV (affirmative answers: Yes = 13.8%, *n* = 1172; No= 68.5%, *n* = 4779; Negative answer: Yes = 86.2%, *n* = 7310; No = 31.5%, *n* = 2198; *X*^2^ = 4849.279, *df* = 2; *p*-value < 0.001) for received information and informed consent requested during procedures. Statistically significant differences were also found for informed consent requested and information received by women according to when they were attended to and the professionals present, except for gynecologists while giving birth (*X*^2^ = 0.006, df = 1; *p*-value = 0.481) and during puerperium (*X*^2^ = 1.925, df = 1; *p*-value = 0.087) (Table 1). Figure 1 shows the distribution of the most representative professionals at all the times when care was received (pregnancy, birth, and puerperium).

### 3.2. Criticized about Their Behavior and Treated with Childish Diminutives in Relation to Professionals, Times, and Areas

We found that 34.5% (*n* = 6045) reported being criticized for their behavior with ironic and discrediting remarks, while 31.4% (*n* = 5502) were treated with nicknames or childish diminutives. With such verbal violence, the most widely identified professionals were midwives with 43.1% (*n* = 2603), gynecologists with 43.8% (*n* = 2646), and nurses with 34.7% (*n* = 2095). The time when such treatment occurred was while giving birth with 68.4% (*n* = 4117) of cases, and the most widely reported area was hospitals with 81.2% (*n* = 4890) of cases.

Statistically significant differences were found in criticizing women according to the type of care received (Public: Yes = 70.6%, *n* = 4269; No = 62.2%, *n* = 6970; Private: Yes = 14.8%, *n* = 892; No = 8.1%, *n* = 810; Mixed: Yes = 14.6%, *n* = 884; No = 29.7%, *n* = 3323; *X*^2^ = 580.115, *df* = 4; *p*-value < 0.001) and perceived OV (affirmative answer: Yes = 68.9%, *n* = 3685; No = 22.0%, *n* = 2247; negative answer: Yes = 31.1%, *n* = 1661; No = 78.0%, *n* = 7698; *X*^2^ = 3292.105, *df* = 2; *p*-value < 0.001). Being treated with childish diminutives also resulted in statistically significant differences for type of care received (Public: Yes = 69.1%, *n* = 3800; No = 62.5%, *n* = 6165; Private: Yes = 12.4%, *n* = 684; No = 9.3%, *n* = 922; Mixed: Yes = 18.5%, *n* = 1018; No = 28.2%, *n* = 2780; *X*^2^ = 204.483, *df* = 4; *p*-value < 0.001) and for perceived OV (affirmative answers: Yes = 54.9%, *n* = 2714; No = 27.1%, *n* = 2443; negative answer: Yes = 45.1%, *n* = 2233; No= 72.9%, *n* = 6562; *X*^2^ = 1135.099, *df* = 2; *p*-value < 0.001).

**Figure 2:**
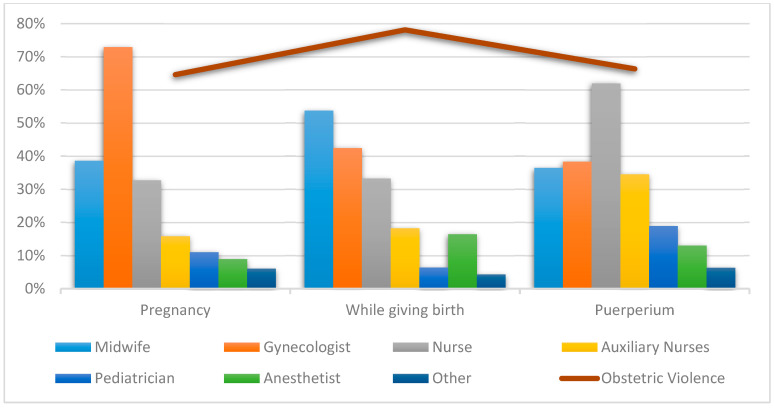
The most representative professionals at all the times care was received in relation to criticized behavior and treated with childish diminutives.

Table 2 and Figure 2 show the distribution of professionals and healthcare areas according to times for being criticized about their behavior and treated with childish diminutives. Statistically significant differences were observed in all the analyzed pairs, except for auxiliary nurses when giving birth (*X*^2^ = 5.232, *df* = 2; *p*-value = 0.073), pediatricians when giving birth (*X*^2^ = 4.091, *df* = 2; *p*-value = 0.129), anesthetists during the puerperium (*X*^2^ = 5.366, *df* = 2; *p*-value = 0.068), and “other” professionals while giving birth (*X*^2^ = 5.678, *df* = 2; *p*-value = 0.058).

### 3.3. Perceiving Coercive Treatment in Relation to Professionals, Areas, and Times

Of our sample, 48.0% (*n* = 8423) stated that they could not resolve their doubts or express their fears or concerns. Of these, 62.7% (*n* = 5257) pointed out gynecologists as the professionals who they could not express themselves to or ask, while 46.0% (*n* = 3853) indicated this problem with midwifes. The most outstanding time was, once again, when giving birth with 66.4% (*n* = 5537) and at hospital with 77.3% (*n* = 6445) of cases.

Statistically significant differences were found for the participants not being able to resolve doubts, or express fears or concerns, according to type of care received (Public: Yes = 73.3%, *n* = 6171; No = 57.1%, *n* = 4920; Private: Yes = 13.4%, *n* = 1129; No = 7.8%, *n* = 668; Mixed: Yes = 13.3%, *n* = 1123; No = 35.2%, *n* = 3030; *X*^2^ = 1148.689, *df* = 4; *p*-value < 0.001) and perceiving OV (affirmative answer: Yes = 66.9%, *n* = 4913; No= 12.2%, *n* = 983; negative answer: Yes = 33.1%, *n* = 2431; No= 87.8%, *n* = 7073; *X*^2^ = 4862.513, *df* = 2; *p*-value < 0.001).

**Figure 3:**
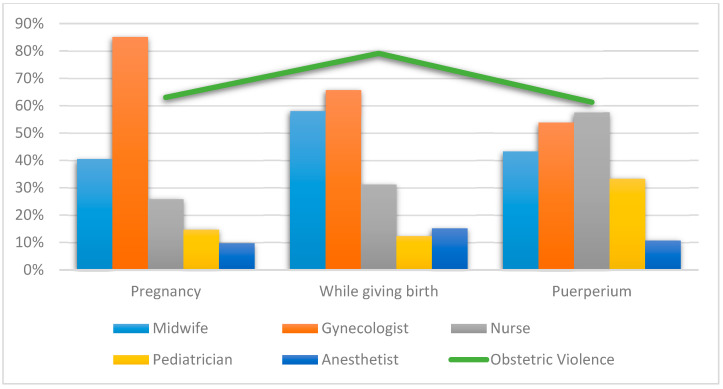
Most representative professionals at each time care was received in relation to not being able to resolve doubts or express fears/concerns.

No statistically significant differences appeared for pediatricians while giving birth (*X*^2^ = 4.213, *df* = 2; *p*-value = 0.122) and anesthetists during puerperium (*X*^2^ = 0.035, *df* = 2; *p*-value = 0.982) for women not being able to resolve doubts or express fears/concerns (Table 3). Figure 3 graphically represents the professionals most reported by women for not being able to resolve doubts or express fears/concerns according to the time when they received care. 

### 3.4. Care Received while Giving Birth and During Puerperium by Hospital Healthcare Professionals

While giving birth, 44.4% (*n* = 7786) of cases perceived that they had undergone unnecessary and/or painful procedures. Of these, 52.3% (*n* = 4026) were neither given reasons nor asked to give consent, while 31.1% (*n* = 2406) were given reasons but not asked to give consent. The healthcare professionals who were women that had indicated they had performed procedures they perceived as painful were 66.9% (*n* = 5148) for midwives and 37.7% (*n* = 5210) for gynecologists. Only 20.2% (*n* = 3323) believed that their birth plan was respected, while 60.2% (*n* = 2313) stated that their birth plan was not respected by gynecologists, and 54.6% (*n* = 2100) stated the same for midwives. The bivariate analyses appear in Table 4 and Table 5.

**Figure 4:**
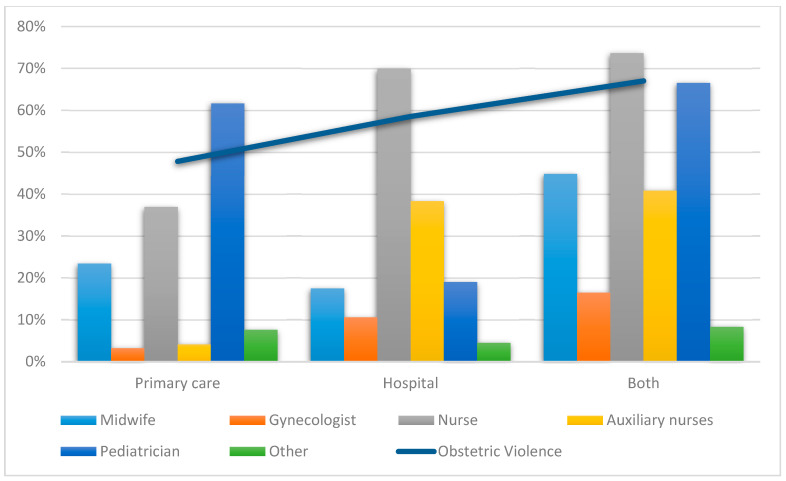
The most represented professionals in all areas in relation to received baby care support.

During puerperium, 39.0% (*n* = 6415) of the women answered not feeling supported in their breastfeeding and baby care decisions, or not noting this support. On this occasion, the most representative professionals were nurses in 60.2% (*n* = 3442) of cases and pediatricians in 37.0% (*n* = 2116) of cases (Figure 4), and at hospital (58.2%, *n* = 3260). For this variable, statistically significant differences were observed for type of care received (Public: Yes = 60.7%, *n* = 6087; No = 74.1%, *n* = 4259; Private: Yes = 8.4%, *n* = 841; No = 12.1%, *n* = 697; Mixed: Yes = 30.9%, *n* = 3099; No = 13.8%, *n* = 795; *X*^2^ = 597.926; *df* = 4; *p*-value < 0.001), and also for perceiving OV (affirmative answer: Yes = 26.9%, *n* = 2480; No= 56.6%, *n* = 2857; negative answer: Yes = 73.1%, *n* = 6734; No = 43.4%, *n* = 2189; *X*^2^ = 1242.229, *df* = 2; *p*-value < 0.001). The bivariate analysis results are shown in Table 6. 

**Figure 5:**
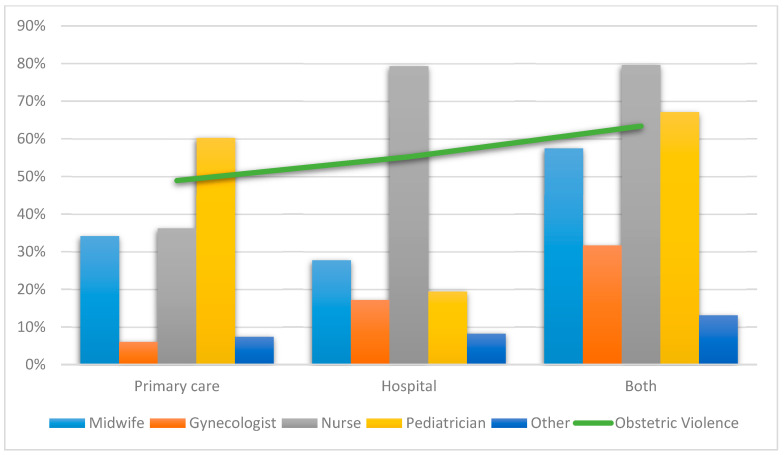
The most represented professionals in all areas in relation to received breastfeeding support.

Of all the women who opted for breastfeeding, 44.3% (*n* = 7219) reported not feeling supported by healthcare professionals or not feeling that this choice was recognized. Nurses (66.8%, *n* = 4112) and pediatricians (38.0%, *n* = 2339) at hospital (56.3%, *n* = 3415) were the most represented professionals and area, respectively (Figure 5). Statistically significant differences were found for type of care received (Public: Yes = 60.0%, *n* = 5450; No = 74.9%, *n* = 4586; Private: Yes = 8.5%, *n* = 769; No = 11.5%, *n* = 705; Mixed: Yes = 31.5%, *n* = 2858; No = 13.6%, *n* = 832; *X*^2^ = 641.731, *df* = 4; *p*-value < 0.001) and for perceiving OV (affirmative answer: Yes = 27.0%, *n* = 2257; No= 55.0%, *n* = 2958; negative answer: Yes = 73.3%, *n* = 6090; No = 45.0%, *n* = 2423; *X*^2^ = 1088.568, *df* = 2; *p*-value < 0.001) (Table 6). 

## 4. Discussion

The present study informs about the main healthcare professionals with whom, areas in which, and times when women perceived more OV. The main variables were analyzed according to the maternity times (pregnancy, giving birth, puerperium) when women perceived OV according to the type of care received, the cluster group established in a previous study [15], and by the involved professionals. The bivariate analysis and its results with cluster groups continued to indicate a certain degree of inequality in the different regions of Spain for OV, which falls in line with previous studies [15,29]. So, we believe that our study results could provide healthcare professionals, organizations, and policies with an idea about those healthcare areas where it would be necessary to seriously evaluate health services related to women’s sexual and reproductive health. After this evaluation, it is important to adapt the care and services offered to women. This effort is needed during women’s sexual and reproductive lives, especially pregnancy, birth, and immediate puerperium, and by always attending to breastfeeding. In line with our study results, this effort should probably be greater in the hospital area.

It is well-known that the birth work and giving birth experience is complex. Moreover, this experience includes subjective psychological and physiological processes. These processes are interrelated and can, in turn, be influenced by social, environmental, organizational, and political contexts [33]. For all these reasons, it is necessary to evaluate these areas in parts to find out how to improve the birth experience and to, thus, increase satisfaction and reduce perceived OV in women while giving birth. Identifying the professionals that women mostly indicate in relation to OV is not an accusing objective of this study. OV is understood to affect women in all healthcare areas, and also in their sexual, physical, emotional, psychological, and affective lives and their relationships. Hence, respecting women’s dignity is key so that OV is not perpetuated. In line with this, the international literature is full of contributions to patients’ dignity, and it has repeatedly demonstrated that nursing is one of the key elements for respecting dignity [34,35], and for fighting for patients’ basic rights and against OV [8].

As the present study shows, OV appears to be a problem in Spain. The fact that such violence is rooted in women’s sexual and reproductive healthcare seems to derive from the way women from any culture and world society have been traditionally treated, which is especially true of Spain [5]. Moreover, the Spanish National Health System has inherited an authoritarian, misogynist, and hierarchized healthcare model, which certainly does not help to eradicate OV from the care that women receive. Some female authors have defended the existence of a training, depersonalization, infantilization process for women, and women being treated as objects, which begins during prenatal visits, continues to the time birth is planned and ends while giving birth [5]. The present study corroborates all these facts but, unlike the definition made by the literature, we can state that, for women, this depersonalization and infantilization process, and women being treated as objects, commences during prenatal visits and ends when giving birth, but puerperium is an important healthcare area that still favors OV when women and their babies receive care. According to the findings herein obtained, in Spain, this fact can be observed at hospitals during the immediate puerperium and later puerperium in the Primary Care area. When the maternity process finishes, the women who have given birth are perceived merely as a body that has had a live baby and who have lived the pregnancy/birth process that is simply another healthcare intervention [5,36], whereas they it should be seen as a physiological process during which healthcare professionals’ main role should be support and accompaniment to maternity and the expected conduct in most cases. We believe that although a change of paradigm in healthcare is starting to take place, we must continue to investigate this in such a wide area in order to accompany this change in scientific evidence, because it can orientate us to secure each step of this change. This viewpoint generates a large future research line which, from our modest point of view, should be complemented in a multidisciplinary manner from medical, nursing, psychological, and even, anthropological areas.

Giving birth in a patriarchal context means that women are more frequently embarrassed and also by anyone who comes into contact with them [1]. Thus, the main healthcare professionals identified by women as being more violent completely coincide with the healthcare professionals involved in maternal and perinatal healthcare in Spain, and in both hospitals and Primary Care. Notwithstanding, the present study found some surprising results, such as identifying professional “anesthetists” during pregnancy. In Spain, it is generally commonplace for women to sign informed consent during a single visit they have with an anesthetist in the event of it being necessary to administer epidural analgesia to women while they give birth. The fact that this single visit comes across as being violent means that we must reflect on how pregnant women are admitted and treated. Moreover, the fact that “midwives” are the most related professionals to perform unnecessary and/or painful procedures allows us to assume that the immense majority of pregnancies and births in our study sample were normal, physiological, and healthy despite not having data to verify this information. Future studies should bear in mind women’s socio-demographic data to verify this hypothesis. 

It is necessary to highlight the “gynecologist” professional because many women identified this healthcare professional in all the analyzed variables, with figures exceeding 85% during pregnancy for variables such as lack of information about informed consent and not being able to resolve doubts or express fears/concerns. Lack of skills and attitudes toward interpersonal communication comes across as a wide gap to bridge in obstetric training. This is why general guidelines to improve obstetric healthcare quality in countries such as Canada highlight communication as a resource set on a central axis [37]. It is also necessary to stress the presence of the professionals that women identified as “other” for each analyzed variable. In healthcare, introducing and identifying oneself to patients should be considered an essential basic communication element to establish a helpful relationship [38]. However, according to the acquired data, it would appear that this is not the case in healthcare in the pregnancy, birth, and puerperium areas in Spain because women were quite unable to identify the “other” person who they perceived did not treat them as they should. One possible assumption deriving from such treatment relates work overload to poor-quality healthcare. Indeed, Spanish healthcare professionals’ work overload is evident. According to the 2019 data provided by the Organisation for Economic Cooperation and Development (OECD), Spain has fewer doctors for every 1000 inhabitants than the mean of other countries belonging to the OECD [39]. If we pay attention to the number of nurses for every 1000 inhabitants, Spain practically occupies the last place for OECD countries with 5.9 nurses for every 1000 inhabitants compared to Norway at the top of this list with 18 nurses for every 1000 inhabitants [39]. Work overload might lead to burnout syndrome, which spells worse quality healthcare for patients [40]. The so-called institutional violence should also be contemplated at this point by means of which the WHO has not only acknowledged that the Health Administration does not spend sufficient human or material resources on friendly healthcare while giving birth [41], but also occupational stress, heavy workload, and lack of accommodation for childbirth areas are factors that perpetuate OV [42]. 

When considering professionals’ training, we need to reflect on the notion that midwife students can learn about how they might act violently while training because this occurs during their training [43]. At the same time, this violence is diluted in a sea of horizontal violence, which is neither reported nor made visible [44]. Too many activists worldwide in general, and in Spain in particular, continue to highlight that healthcare studies that contemplate the gender data gaps that exist for diseases and treatments and psychological processes, such as pregnancy, birth, or menopause, are still lacking [36]. This gender data gap has clearly been identified in the scientific literature in the medical text books and curricular syllabi of some Health Sciences faculties [45,46,47]. This also takes place in Spain if we consider that the description of physiological birth in a non-pathological sense has not been included in the manuals of the medical specialties studied in Spanish universities until the present century [36]. Thus, future research lines addressing health professionals’ training and their working conditions seem important. In parallel, the study by Ravaldi et al. [48] reveals that women expect professionalism, humanity, and empathy, and the capacity to listen and attend to their requirements, as regards the quality of the care they receive while giving birth. So, restructuring Spanish health services is necessary by heeding international recommendations about the need to provide more healthcare staff members to improve healthcare quality, attend to women’s physiological processes (e.g., pregnancy and childbirth) as part of educational curricula, and pay special attention to communication skills. Future studies that contemplate different educational interventions with healthcare professionals should confirm short- and long-term changes in attitude and skills in perinatal healthcare. 

Healthcare quality assessments during the perinatal period, regardless of being made with indicators of satisfaction with healthcare and birth delivery or OV, should be relevant for healthcare professionals, administrators, organizations, and politicians so they can make decisions about suitable changes to improve healthcare quality. 

This study has its limitations, which should be taken to account when interpreting its results. It is first necessary to consider that non-probabilistic sampling was performed, which could affect the sample’s representativeness. A certain selection bias could have come into play because the questionnaire was distributed into groups that could have been more sensitive to the studied theme such as «Birth is Ours». It is worth noting that some variables were not included, such as age, cultural/socio-economic variables, number of children, or date of birth, to perform a descriptive socio-demographic analysis to make comparisons with other populations. Future studies should take this fact into account. Our study is a retrospective one based on how women perceive OV. This might involve memory and information biases despite scientific evidence for the perinatal period being well-retained in women’s memory [49]. 

We also stress that the Spanish healthcare model and its birth care shape an almost unique healthcare model, which means that some of its results cannot be extrapolated to other health systems. Finally, we highlight that some female authors, who have assessed birth satisfaction and the existing scales to measure it, criticize the fact that those instruments that allow both the mother’s and couple’s satisfaction to be jointly assessed are lacking [26]. Nor does the present study contemplate how the partner or accompanying person perceives OV, which is another increasingly interesting future research field. 

## 5. Conclusions

The present study reflected on the main professionals with whom OV occurs in Spanish centers, namely midwives and gynecologists. Surprisingly, it also identified other healthcare professionals such as anesthetists or “other”. This poses a basic training problem in physiological processes for women’s sexual and reproductive health and communication skills, which are two keys to be considered to bring about a true change in paradigm. 

Time of birth and hospitals were also the most frequent areas that women identified where OV peaked. Therefore, it is necessary to truly reflect on the care received during the perinatal period in hospitals with a view to improve the care received at this time. 

Finally, it is necessary to also reflect on the Spanish National Health System’s structure and management because they might result in its healthcare professionals’ work overload, and to such an extent that it is very difficult to improve healthcare quality without making more organizational or political interventions other than only educational ones. Finally, it is necessary to place more pressure on raising standards for the numbers of healthcare members of staff in Spain to equal or be similar to those of other OECD countries.

## Figures and Tables

**Table 1:** Informed consent requested and information received depending on the times when care was received, the professionals present, and healthcare areas.

	Time Care Was Received								
	Pregnancy			While Giving Birth			Puerperium		
	*n*	%	*p* ^1^	*n*	%	*p* ^1^	*n*	%	*p* ^1^
Professional									
Midwife	1366	44.9	<0.001	3816	59.0	<0.001	974	58.9	<0.001
Gynecologist	2612	85.8	<0.001	4785	74.0	0.481	1245	75.3	0.087
Nurse	959	31.5	0.007	2070	32.0	<0.001	966	58.4	<0.001
Pediatrician	606	16.6	<0.001	944	14.6	<0.001	643	38.9	<0.001
Anesthetist	525	17.2	0.002	1210	18.7	<0.001	396	24.0	<0.001
Other	161	5.3	<0.001	208	3.2	<0.001	102	6.2	<0.001
Area									
Primary care	393	12.9		28	0.4		75	4.5	
Hospital	1766	57.9	<0.001	5536	85.6	<0.001	999	60.5	<0.001
Both areas	899	29.2		904	14.0		245	35.0	
Cluster Group									
1	1152	37.8		2444	37.8		605	36.6	
2	902	29.6		1931	29.8		472	28.6	
3	475	15.6	0.246	1072	16.6	0.671	296	17.9	0.224
4	505	16.6		996	15.4		273	16.5	
5	17	0.6		30	0.5		7	0.4	
Type of care received									
Public	2321	76.1		4843	74.8		1254	75.9	
Private	458	15.0	<0.001	872	13.5	0.235	276	16.7	<0.001
Mixed	272	8.9		758	11.7		123	7.4	
Perceiving OV ^2^									
Yes	1746	65.1	<0.001	4160	74.2	<0.001	1021	71.6	<0.001
No	936	34.9		1450	25.8		404	28.4	

^1^ Chi-squared test or Fisher’s exact test; ^2^ OV: obstetric violence.

**Table 2:** Criticized behavior and treated with childish diminutives according to times, professionals, and areas.

	Time Care Was Received								
	Pregnancy			While Giving Birth			Puerperium		
	*n*	%	*p* ^2^	*n*	%	*p* ^2^	*n*	%	*p* ^2^
Professional									
Midwife	720	38.6	<0.001	2210	53.7	<0.001	652	36.4	<0.001
Gynecologist	1356	72.9	<0.001	1746	42.4	0.001	686	38.3	<0.001
Nurse	609	32.7	0.010	1366	33.2	<0.001	1109	62.0	<0.001
AN ^1^	295	15.8	0.003	749	18.2	0.073	617	34.5	<0.001
Pediatrician	205	11.0	<0.001	264	6.4	0.129	338	18.9	<0.001
Anesthetist	165	8.9	<0.001	676	16.4	<0.001	232	13.0	0.068
Other	112	6.0	<0.001	175	4.3	0.058	112	6.3	<0.001
Area									
Primary care	311	16.7	<0.001	5	0.1		160	8.9	
Hospital	1024	55.1		3600	87.6	<0.001	1171	65.5	<0.001
Both areas	524	28.2		503	12.2		457	25.6	
Cluster Group									
1	736	39.5		1560	37.9		672	37.5	
2	513	27.5		1206	29.3		511	28.5	
3	300	16.1	0.436	675	16.4	0.169	292	16.3	0.589
4	305	16.4		664	16.1		310	17.3	
5	10	0.5		12	0.3		6	0.3	
Type of care received									
Public	1298	69.6		2877	69.9		1314	73.4	
Private	306	16.4	0.048	689	16.7	<0.001	261	14.6	0.001
Mixed	260	13.9		551	13.4		216	12.1	
Perceiving OV ^3^									
Yes	1064	64.6	<0.001	2881	78.1	<0.001	1038	66.3	0.003
No	582	35.4		809	21.9		528	33.7	

^1.^ AN: auxiliary nurses; ^2^ Chi-squared test or Fisher’s exact test; ^3^ OV: obstetric violence.

**Table 3:** Not resolving doubts or expressing fears/concerns at the time they received care, professionals, and areas.

	Time Care Was Received								
	Pregnancy			While Giving Birth			Puerperium		
	*n*	%	*p* ^1^	*n*	%	*p* ^1^	*n*	%	*p* ^1^
Professional									
Midwife	1247	40.5	<0.001	3215	58.1	<0.001	1211	43.3	0.001
Gynecologist	2624	85.1	<0.001	3641	65.8	<0.001	1506	53.8	<0.001
Nurse	795	25.8	<0.001	1723	31.2	0.038	1613	57.6	<0.001
Pediatrician	454	14.7	<0.001	681	12.3	0.122	932	33.3	<0.001
Anesthetist	298	9.7	0.008	843	15.2	<0.001	298	10.7	0.982
Area									
Primary care	490	15.9	<0.001	9	0.2		270	9.7	
Hospital	1682	54.7		4598	83.4	<0.001	1727	61.8	<0.001
Both	905	29.4		904	16.4		798	26.8	
Cluster Group									
1	1179	38.2		2063	37.3		1054	37.6	
2	886	28.6		1666	30.1		826	29.5	
3	501	16.2	0.628	910	16.4	0.137	475	16.9	0.717
4	508	16.5		872	15.7		431	15.4	
5	17	0.6		26	0.5		17	0.6	
Type of care received									
Public	2278	73.8		4073	73.6		2114	75.4	
Private	444	14.4	0.006	854	15.4	<0.001	356	12.7	0.010
Mixed	366	11.9		610	11.0		333	11.9	
Perceiving OV ^2^									
Yes	1701	63.0	<0.001	3842	79.1	<0.001	1486	61.3	<0.001
No	998	37.0		1016	20.9		938	38.7	

^1^ Chi-squared test or Fisher’s exact test; ^2^ OV: obstetric violence.

**Table 4:** Bivariate analysis about the birth-related variables.

	Yes		No		Not Know/No Answer				
	*n*	%	*n*	%	*n*	%	*X* ^2^	*df* ^2^	*p* ^3^
**Perceiving Unnecessary and/or Painful Procedures**									
Professional									
Midwife	4549	68.4	1	100.0	598	57.2	84.884	4	<0.001
Gynecologist	4576	68.8	1	100.0	634	60.6	70.128	4	<0.001
Nurse	2487	37.4	-	-	282	36.5	59.055	4	<0.001
Pediatrician	1074	16.2	-	-	125	12.0	65.998	4	<0.001
Other	319	4.8	-	-	38	3.6	59.688	4	<0.001
Cluster Group									
1	2930	37.6	3494	40.3	405	39.4			
2	2318	29.8	2511	28.9	291	26.9			
3	1281	16.5	1365	15.7	202	18.7	23.812	8	0.002
4	1223	15.7	1250	14.4	157	14.5			
5	34	0.4	55	0.6	5	0.5			
Type of care received									
Public	5561	71.4	5092	58.7	797	73.8			
Private	1069	13.7	690	8.0	71	6.6	850.740	4	<0.001
Mixed	1156	14.8	2893	33.3	212	19.6			
Perceiving OV ^1^									
Yes	5077	74.3	697	8.4	277	39.5	6862.819	2	<0.001
No	1753	25.7	7555	91.6	424	60.5			
**Informed Consent Requested**									
Cluster Group									
1	183	37.6	2636	37.9	97	38.6			
2	127	26.1	2061	29.6	69	27.5			
3	98	20.1	1153	16.6	44	17.5	7.362	8	0.498
4	75	15.4	1079	15.5	40	15.9			
5	4	0.8	30	0.4	1	0.4			
Type of care received									
Public	343	70.4	5151	74.0	179	71.3			
Private	23	4.7	866	12.4	24	9.6	69.316	4	<0.001
Mixed	121	24.8	942	13.5	48	19.1			
Perceiving OV ^1^									
Yes	136	32.9	4633	78.5	108	59.3	456.102	2	<0.001
No	278	67.1	1267	21.5	74	40.7			

^1^ OV: obstetric violence; ^2^ *df*: degrees of freedom; ^3^ Chi-squared test.

**Table 5:** Bivariate analysis about birth plan-related variables.

	Did Not Hand In		Yes		No, but I Know the Reasons		No, and I Don’t Know the Reasons		NK/NA ^1^				
	*n*	%	*n*	%	*n*	%	*n*	*%*	*n*	*%*	*X* ^2^	*df* ^3^	*p* ^4^
Professional													
Midwife	-	-	-	-	655	37.9	1445	68.4	-	-	417.466	2	<0.001
Gynecologist	-	-	-	-	924	53.4	1389	65.7	-	-	210.951	2	<0.001
Nurse	-	-	-	-	171	9.9	637	30.1	-	-	375.517	2	<0.001
Pediatrician	-	-	-	-	56	3.2	195	9.2	-	-	246.050	2	<0.001
Other	-	-	-	-	99	5.7	105	5.0	-	-	212.966	2	<0.001
Cluster Group													
1	3568	40.7	1299	39.1	642	37.0	752	35.6	178	35.8			
2	2486	28.3	971	29.2	545	31.4	641	30.3	150	30.2			
3	1381	15.7	518	15.6	293	16.9	384	18.2	96	19.3	37.730	16	0.003
4	1286	14.7	516	15.5	248	14.3	329	15.6	70	14.1			
5	51	0.6	19	0.6	8	0.5	9	0.4	3	0.6			
Type of care received													
Public	5857	66.8	1870	56.3	1254	72.2	1516	71.7	314	63.2			
Private	838	9.6	235	7.1	84	4.8	389	18.4	64	12.9	692.005	8	<0.001
Mixed	2073	23.7	1218	36.7	398	22.9	210	9.9	119	23.9			
Perceiving OV ^2^													
Yes	3022	38.9	270	8.4	567	37.0	1572	82.6	160	40.5	2806.912	4	<0.001
No	4747	61.1	2937	91.6	965	63.0	332	17.4	235	59.5			

^1^ NK/NA: Not know/No Answer; ^2^ OV: obstetric violence; ^3^ *df*: degrees of freedom; ^4^ Chi-squared test.

**Table 6:** Treatment during puerperium according to professionals and areas.

	Area								
	Primary care		Hospital		Both				
	*n*	%	*n*	%	*n*	%	*X* ^2^	*df* ^2^	*p* ^3^
**Support Baby Care**									
Professional									
Midwife	367	23.4	568	17.5	347	44.9	280.608	4	<0.001
Gynecologist	50	3.2	344	10.6	127	16.5	147.204	4	<0.001
Nurse	579	36.9	2276	69.9	568	73.6	642.048	4	<0.001
AN ^1^	64	4.1	1248	38.3	315	40.8	717.018	4	<0.001
Pediatrician	970	61.7	620	19.0	513	66.5	1148.343	4	<0.001
Other	120	7.6	146	4.5	64	8.3	47.969	4	<0.001
Cluster Group									
1	623	39.6	1162	35.6	311	40.2			
2	468	29.8	925	28.4	197	25.5			
3	252	16.0	593	18.2	124	16.0	21.023	8	0.007
4	226	14.2	567	17.4	138	17.9			
5	7	0.4	13	0.4	3	0.4			
Type of care received									
Public	1199	76.2	2366	72.6	580	75.0			
Private	139	8.8	434	13.3	104	13.5	25.505	4	<0.001
Mixed	235	14.9	460	14.1	89	11.5			
Perceiving OV ^4^									
Yes	656	47.8	1673	58.5	458	67.0	77.301	2	<0.001
No	716	52.2	1186	41.5	226	33.0			
**Support Breast Feeding**									
Professional									
Midwife	569	34.2	947	27.8	567	57.6	301.616	4	<0.001
Gynecologist	101	6.1	586	17.2	312	31.7	302.730	4	<0.001
Female nurse	603	36.2	2705	79.3	784	79.6	1095.350	4	<0.001
Pediatrician	1005	60.3	663	19.4	661	67.1	1207.195	4	<0.001
Other	123	7.4	281	8.2	129	13.1	33.841	4	<0.001
Cluster Group									
1	658	39.5	1226	35.9	370	37.5			
2	497	29.5	972	28.5	296	30.0			
3	277	16.6	615	18.0	165	16.7	15.859	8	0.044
4	227	13.6	585	17.1	149	15.1			
5	8	0.5	17	0.5	6	0.6			
Type of care received									
Public	1279	76.7	2500	73.2	761	77.2			
Private	155	9.3	407	11.9	131	13.3	29.996	4	<0.001
Mixed	233	14.0	508	14.9	94	9.5			
Perceiving OV ^4^									
Yes	717	48.9	1654	55.2	553	63.4	47.174	2	<0.001
No	750	51.1	1340	44.8	319	36.6			

^1^ AN: auxiliary nurses; ^2^ df: degrees of freedom; ^3^ Chi-squared test or Fisher’s exact test; ^4^ OV: obstetric violence.

## Data Availability

The data presented in this study are available on request from the corresponding author.
